# TGF-β1-mediated repression of SLC7A11 drives vulnerability to GPX4 inhibition in hepatocellular carcinoma cells

**DOI:** 10.1038/s41419-020-2618-6

**Published:** 2020-05-29

**Authors:** Do Hyung Kim, Won Dong Kim, Sang Kyum Kim, Dae Hyuk Moon, Seung Jin Lee

**Affiliations:** 1 College of Pharmacy, Chungnam National University, 99 Daehak-ro, Yuseong-gu, Daejeon, 34134 Republic of Korea; 2 Convergence Medicine Research Center, Asan Institute for Life Sciences, Asan Medical Center, 88 Olympic-ro 43-gil, Songpa-gu, Seoul, 05505 Republic of Korea; 3 Department of Nuclear Medicine, Asan Medical Center, University of Ulsan, College of Medicine, 88 Olympic-ro 43-gil, Songpa-gu, Seoul, 05505 Republic of Korea

**Keywords:** Targeted therapies, Cell death, Pharmacodynamics

## Abstract

System x_c_^−^ contributes to glutathione (GSH) synthesis and protects cells against ferroptosis by importing cystine and exchanging it with glutamate. Transforming growth factor β1 (TGF-β1) induces redox imbalance; however, its role in system x_c_^−^ regulation remains poorly understood. The present study was the first to show that TGF-β1 repressed the protein and mRNA levels of xCT, a catalytic subunit of system x_c_^−^, in PLC/PRF/5, Huh7, Huh6, and HepG2 cells with an early TGF-β1 gene signature but not in SNU387, SNU449, SNU475, and SK-Hep1 cells with a late TGF-β1 gene signature. TGF-β1 treatment for 24 h reduced xCT expression in a dose-dependent manner but this TGF-β1-induced repression was blunted by pretreatment with a TGF-β1 receptor inhibitor. TGF-β1-mediated xCT repression was prevented by Smad3, but not Smad2 or Smad4, knockdown, whereas it was enhanced by Smad3 overexpression. TGF-β1 decreased GSH levels in control cells but not xCT-overexpressed cells. Furthermore, TGF-β1 increased reactive oxygen species (ROS) levels in PLC/PRF/5 cells and enhanced tert-butyl hydroperoxide-induced ROS levels in Huh7 cells; these changes were reversed by xCT overexpression. TGF-β1 treatment ultimately induced the ferrostatin-1- and deferoxamine-dependent lipid peroxidation after 2 days and 8 days in PLC/PRF/5 and Huh7 cells but not in SNU475 and SK-Hep1 cells. Pre-treatment of TGF-β1 for 2 days enhanced the reduction of cell viability induced by RSL3, a GSH peroxidase 4 (GPX4) inhibitor, in PLC/PRF/5 and Huh7 cells. In conclusion, TGF-β1 represses xCT expression via Smad3 activation and enhances lipid peroxidation in hepatocellular carcinoma cells with an early TGF-β1 signature, which would benefit from the targeting of GPX4.

## Introduction

System x_c_^−^, a cystine/glutamate exchange transporter, uptakes cystine driven by higher concentrations of glutamate in the cytoplasm^1^. Cystine is reduced to cysteine and then used as a building block during the biosynthesis of glutathione (GSH), which is a substrate for GSH peroxidase 4 (GPX4) that protects cells against lipid peroxidation^1–3^. Inhibition of system x_c_^−^ promotes the iron-mediated accumulation of lipid peroxidation end products and ultimately induces ferroptosis, which is a form of cell death distinct from apoptosis and necroptosis at the morphological, biochemical, and genetic levels^4^. Some populations of cancer cells are highly dependent on system x_c_^−^ or GPX4 to cope with the redox imbalance caused by their rapid growth and the subsequent limited availabilities of oxygen and nutrients^5^. For example, triple-negative breast tumors are highly dependent on system x_c_^−^ for survival and become glutamate auxotrophs^6^. Similarly, therapy-resistant mesenchymal cells are dependent on the lipid peroxidase pathway, which protects cells against ferroptosis^7^.

System x_c_^−^ comprises the SLC7A11 catalytic subunit, which is also called xCT, and the SLC3A2 non-catalytic heavy subunit, which is known as 4F2hc^1^. xCT expression is correlated with system x_c_^−^ activity and poor prognoses for various types of tumors, including hepatocellular carcinoma (HCC), colorectal cancer, and glioblastoma^8–10^. xCT expression is tightly regulated by signaling pathways that control cancer hallmarks. For example, epidermal growth factor receptor^11^ and CD44 variants^12^ directly interact with xCT and stabilize its expression at the cell surface, which enhances antioxidant capacity. In contrast, oncogenic PI3KCA inhibits xCT and promotes methionine dependency in mammary epithelial tumors^13^, and 5′ adenosine monophosphate-activated protein kinase (AMPK)-mediated phosphorylation of Beclin-1 promote ferroptosis via complexation with xCT^14^. xCT expression is transcriptionally activated by nuclear factor erythroid 2-related factor 2 (Nrf2) and activating transcription factor 4 (ATF4) under conditions of oxidative stress or hypoxia^15,16^, whereas it is inhibited by the tumor suppressor TP53 following reactive oxygen species (ROS)-induced stress independent of its functions regulating apoptosis and senescence^17^. Taken together, these findings indicate that xCT expression is precisely regulated by various growth-regulating signals to meet genetic, metabolic, and environmental needs.

Transforming growth factor β1 (TGF-β1) is a dichotomous cytokine that acts as a tumor suppressor in low-grade carcinoma cells but as a promotor of metastasis in advanced carcinoma cells^18^. Active TGF-β1 dimers bind to TGF-β1 receptor II and trigger the cross-phosphorylation of TGF-β1 receptor I, which, in turn, propagates canonical signals mediated by Smad proteins or non-canonical pathways involving Rho, c-Jun N-terminal kinase (JNK), and RhoA-Rho-associated kinase (ROCK). ROS generation by TGF-β1 plays important roles in the induction of apoptosis or metastasis^19^. The principle source of ROS by TGF-β1 is nicotinamide adenosine dinucleotide phosphate (NADPH) oxidase-4 (NOX4), which generates superoxide anions by transporting electrons from cytosolic NADPH across biological membranes to molecular oxygen sources^20,21^. NOX4 upregulation is required for TGF-β1-induced apoptosis, as well as senescence in hepatocytes and some HCC cells^22,23^. Similar to NOX4, TGF-β1 downregulates glutamate cysteine ligase catalytic (GCLC) subunits in mammary epithelial cells and glutathione-S-transferase (GST) in primary hepatocytes^19^. However, it remains unclear whether these TGF-β1-mediated redox imbalances are mediated by system x_c_^−^ and lead to the accumulation of lipid peroxidation end products.

Therefore, in the present study, we assessed whether system x_c_^−^ was involved in TGF-β1-mediated ROS generation in HCC cell lines. The results revealed that TGF-β1 regulated xCT expression, sensitized cells to lipid peroxidation, and ultimately provided a vulnerability to a GPX4 inhibitor in HCC cell lines in which TGF-β1-induced cytostasis.

## Results

### TGF-β1 repressed xCT expression in HCC cell lines with an early TGF-β1 gene signature

**Fig. 1:**
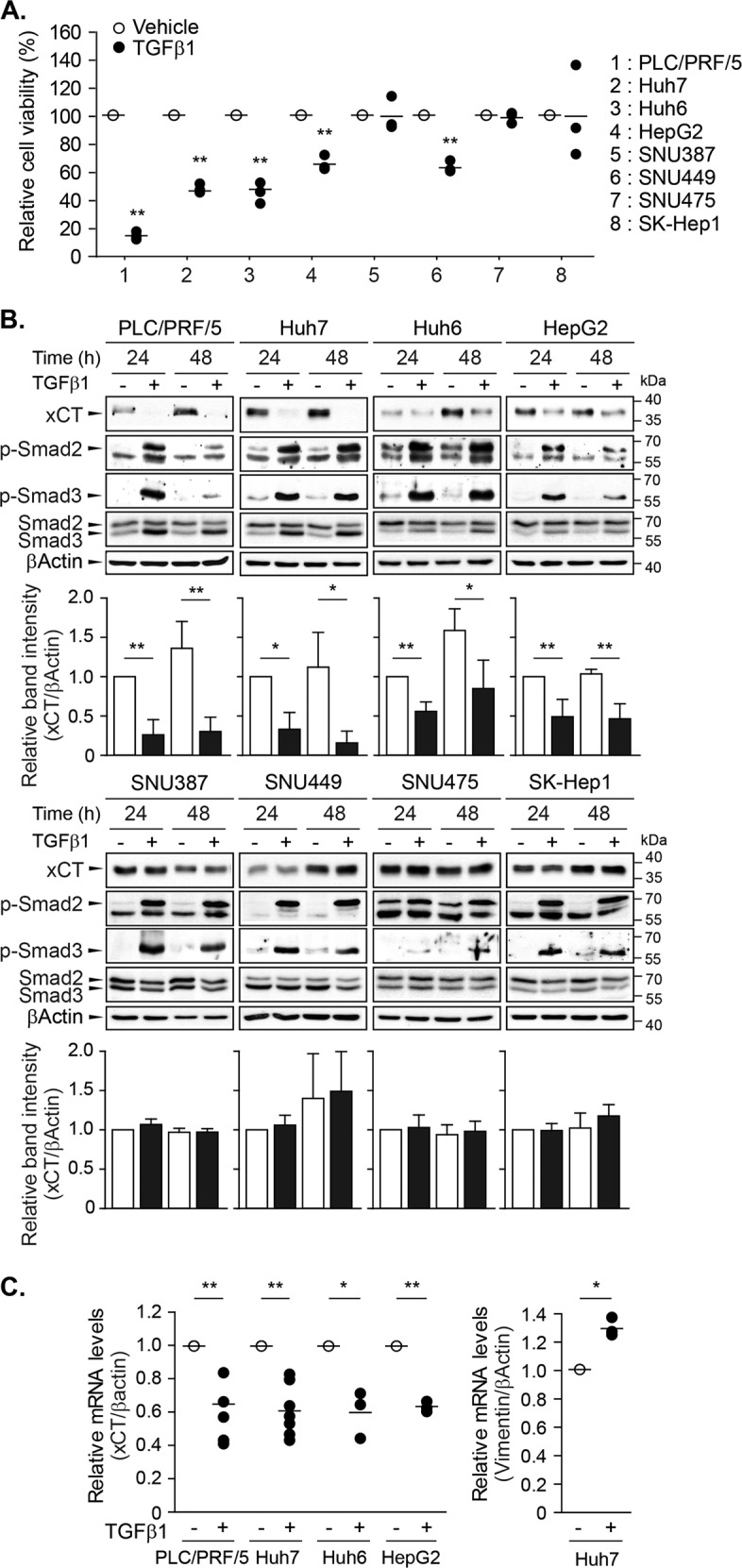
The repression of xCT expression by TGF-β1 in a subset of HCC cell lines. **a** TGF-β1 was administered to PLC/PRF/5, Huh7, Huh6, HepG2, SNU387, SNU449, SNU475, and SK-Hep1 cells at a concentration of 5 ng/mL and cell viability was measured using the CellTiter Glo® assay after 6 days; three independent experiments were performed in triplicate. **b** TGF-β1 was applied to PLC/PRF/5 (*n* = 4), Huh7 (*n* = 3), Huh6 (*n* = 4), HepG2 (*n* = 4), SNU387 (*n* = 3), SNU449 (*n* = 3), SNU475 (*n* = 3), and SK-Hep1 (*n* = 3) cells for 24 or 48 h at a concentration of 5 ng/mL, and then whole-cell lysates were assessed with western blot analyses. Representative images are shown. **c** PLC/PRF/5 (*n* = 5), Huh7 (*n* = 7), Huh6 (*n* = 3), and HepG2 (*n* = 3) cells were treated with 5 ng/mL of TGF-β1 for 24 h and then collected for RT-PCR analyses to detect the mRNA levels of xCT, vimentin, and β-actin; vimentin was used as a positive TGF-β1-responsive gene. All data are expressed as the mean ± SD. ^*^P < 0.05, ^**^P < 0.01, TGF-β1 treatment versus vehicle at each timepoint or in each cell line.

A previous comparative genomics study discriminated two subtypes of human HCC cell lines based on TGF-β1 gene signatures^24^: PLC/PRF/5, Huh7, Huh6, and HepG2 cells were characterized by an early TGF-β1 gene signature associated with TGF-β1-induced cytostasis and apoptosis; and SNU387, SNU449, SNU475, and SK-Hep1 cells were defined by a late TGF-β1 gene signature lacking TGF-β receptors, and exhibited high levels of epithelial-to-mesenchymal transition (EMT)-associated proteins. We confirmed that treatment with 5 ng/mL of TGF-β1 for 6 days inhibited the growth of PLC/PRF/5, Huh7, Huh6, and HepG2 cells (Fig. 1a), as previously reported^21,24^. Although SNU449 cells are classified as having a late TGF-β1 gene signature^24^, they exhibited cytostasis in the present and previous results^25^ presumably because they may share some aspects of both TGF-β1 gene signatures.

To determine whether TGF-β1 regulated system x_c_^−^, the effects of TGF-β1 on xCT expression were examined in these two subtypes of cell lines. Treatment with 5 ng/mL of TGF-β1 for 24 h significantly reduced xCT expression in PLC/PRF/5, Huh7, Huh6, and HepG2 cell lines by 74, 67, 44, and 51%, respectively; this repression was still observed 48 h after treatment. However, TGF-β1 did not induce significant changes in the SNU387, SNU449, SNU475, and SK-Hep1 cell lines (Fig. 1b). The levels of phosphorylated Smad2 and Smad3 were increased by TGF-β1 in all tested cell lines, whereas Smad3 expression was induced by TGF-β1 only in PLC/PRF/5, Huh7, Huh6, and HepG2 cells. Treatment with 5 ng/mL of TGF-β1 for 24 h decreased the mRNA levels of xCT to 64.5, 60.2, 59.3, and 62.4% of vehicle treatment in PLC/PRF/5, Huh7, Huh6, and HepG2 cells, respectively (Fig. 1c). TGF-β1 enhanced vimentin mRNA expression, which was used as a positive control in response to TGF-β1. These results indicated that TGF-β1 repressed xCT expression in HCC cell lines with an early TGF-β1 gene signature, which may be mediated by transcriptional regulation, at least in part.

### TGF-β1 repressed xCT expression in time- and dose-dependent manners

**Fig. 2:**
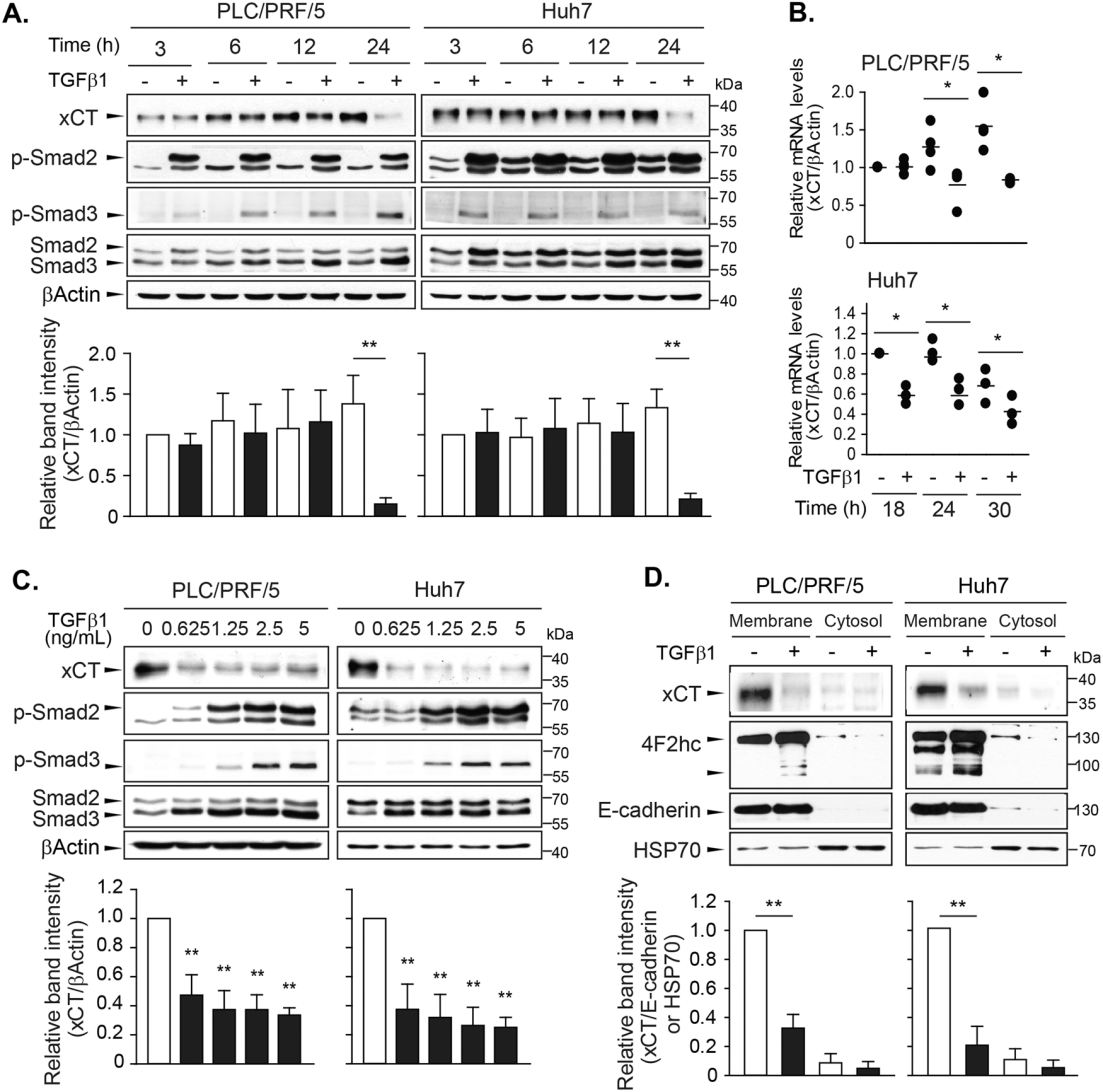
Time- and dose-dependent repressions of xCT expression by TGF-β1 in PLC/PRF/5 and Huh7 cells. Cells were treated with TGF-β1 at a concentration of 5 ng/mL for 3, 6, 12, or 24 h and sampled for western blot analysis (**a**) or for 18, 24, or 30 h and sampled for RT-PCR analyses (**b**). **c** TGF-β1 was administered at the concentrations of 0, 0.625, 1.25, 2.5, and 5 ng/mL for 24 h and whole-cell lysates were used for western blot analysis. **d** PLC/PRF/5 and Huh7 cells treated with 5 ng/mL of TGF-β1 for 24 h were fractionated to obtain plasma membrane and cytosol samples and then used for Western blot analysis. E-cadherin and HSP70 were assessed as marker proteins for the plasma membrane and cytosol fractions, respectively. Representative data from three independent experiments are shown. *P < 0.05, **P < 0.01, TGF-β1 versus vehicle control.

The effects of TGF-β1 on xCT expression were further examined in the PLC/PRF/5 and Huh7 cell lines, which exhibited the highest and moderate degrees of inhibition of cell viability in response to TGF-β1, respectively. Treatment with 5 ng/mL of TGF-β1 for 3, 6, 12, and 24 h increased Smad2 and Smad3 phosphorylation beginning at 3 h and Smad3 expression beginning at 12 h (Fig. 2a). At 24 h, TGF-β1 significantly repressed xCT expression by 89.1% in PLC/PRF/5 cells and by 84.2% in Huh7 cells. Significant decrease in mRNA level of xCT was observed from 24 h in PLC/PRF/5 cells and from 18 h in Huh7 cells (Fig. 2b). Treatment with TGF-β1 at different doses for 24 h revealed significant dose-dependent decreases in xCT expression with Smad2/3 phosphorylation and Smad3 induction beginning at concentrations of 0.625 ng/mL (Fig. 2c).

xCT was primarily located in the plasma membrane fraction (Fig. 2d) with the heavy chain of system x_c_^−^, i.e., 4F2hc. Treatment with 5 ng/mL of TGF-β1 reduced xCT levels in the plasma membrane fraction, whereas there were no changes in 4F2hc levels, which suggested that TGF-β1 selectively regulated the catalytic subunit of system x_c_^−^. E-cadherin and heat shock protein 70 (HSP70) were detected as membrane and cytosol markers, respectively. Taken together, these results indicate that TGF-β1 at physiologically relevant concentration decreased xCT expression in cytosol and membrane fraction following Smad2/3 activation.

### Smad3 downstream of the TGFβ receptor mediated xCT repression

**Fig. 3:**
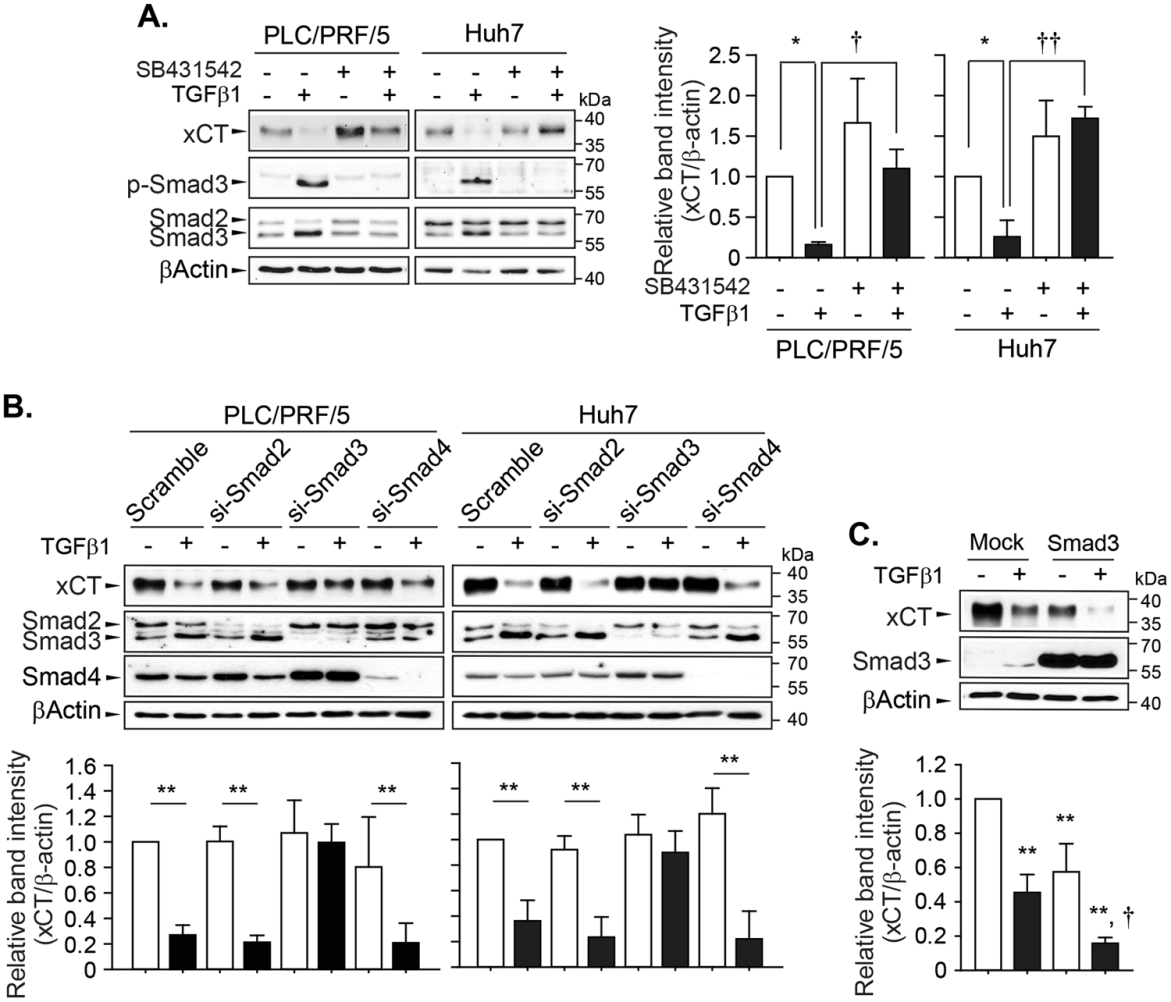
Canonical TGF-β1 signaling pathways involved in the regulation of xCT expression. **a** PLC/PRF/5 and Huh7 cells were pretreated with 2.5 µM of SB431542 for 1 h and then challenged with 5 ng/mL of TGF-β1 for 24 h (*n* = 3). **b** PLC/PRF/5 and Huh7 cells were transiently transfected with scrambled siRNA or siRNA targeting Smad2 (*n* = 4), Smad3 (*n* = 3 for PLC/PRF/5 cells and *n* = 6 for Huh7 cells), or Smad4 (*n* = 3) and then exposed to TGF-β1 for 24 h. **c** Huh7 cells (*n* = 3) were transiently transfected with mock- or Smad3-expressing plasmid, recovered overnight, and then further incubated with TGF-β1 for 24 h; total cell lysates were used for the western blot analysis. Data from independent experiments are expressed as the mean ± SD; representative images are shown. *P < 0.05, **P < 0.01, TGF-β1 versus vehicle control; †*P* < 0.05, ††*P* < 0.01, TGF-β1 alone versus TGF-β1 with SB431542 or TGF-β1 in mock-transfected cells versus TGF-β1 in xCT-transfected cells.

Next, we investigated the mechanisms underlying TGF-β1-mediated xCT repression. The inhibition of TGF-β1 receptor I by pretreatment with 2.5 µM of SB431542 prevented the decrease in xCT expression levels induced by exposure to 5 ng/mL of TGF-β1 for 24 h in PLC/PRF/5 and Huh7 cells (Fig. 3a). Specifically, SB431542 completely inhibited the TGF-β1-induced phosphorylation and induction of Smad3. TGF-β1 also repressed xCT expression in control cells and Smad2- or Smad4-knockdown cells but failed to do so in Smad3-knockdown cells (Fig. 3b); the specific knockdown of Smad2, Smad3, or Smad4 was confirmed in the same samples. Smad3 overexpression in Huh7 cells reduced xCT expression to a similar extent as TGF-β1 (Fig. 3c), while TGF-β1 treatment further decreased xCT expression in cells transfected with Smad3. These results suggested that activation of the TGF-β1 receptor and Smad3 were required for the regulation of xCT by TGF-β1 in these cells.

**Fig. 4:**
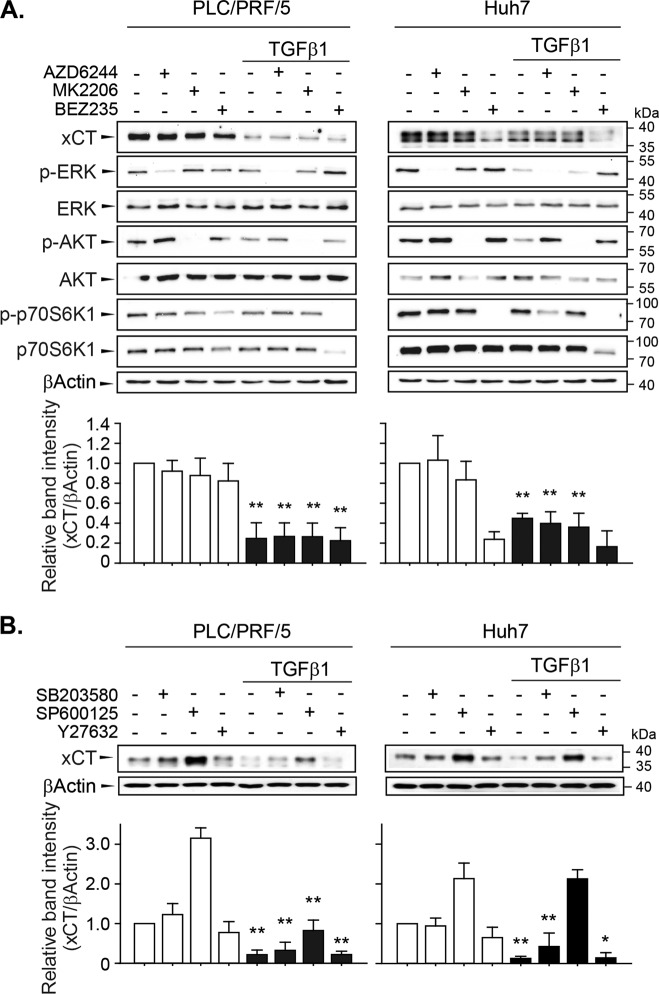
Non-canonical TGF-β1 signaling pathways involved in the regulation of xCT expression. **a** PLC/PRF/5 (*n* = 5) and Huh7 (*n* = 4) cells were pretreated with AZD6244 (0.1 µM for PLC/PRF/5 cells and 1 µM for Huh7 cells), MK2206 (0.1 µM for PLC/PRF/5 cells and 1 µM for Huh7 cells), or BEZ235 (0.01 µM for PLC/PRF/5 cells and 0.1 µM for Huh7 cells) for 1 h and then further incubated with TGF-β1 for 24 h. **b** PLC/PRF/5 (*n* = 3) and Huh7 (*n* = 3) cells were pretreated with either SB203580 (5 µM), SP600125 (10 µM), or Y27632 (10 µM) for 1 h and then further incubated with TGF-β1 for 24 h; total cell lysates were used for western blot analysis. Independent experiments are expressed as the mean ± SD; representative images are shown. *P < 0.05, **P < 0.01, TGF-β1 versus vehicle treatment in each of the inhibitor-treated cells.

TGF-β1 can transmit non-canonical signals, including via the Ras/Raf/MEK, phosphoinositide-3-kinase (PI3K)/protein kinase B (AKT)/mammalian target of rapamycin (mTOR), p38 mitogen-activated protein kinase (MAPK)/JNK, and RhoA/ROCK pathways^26^. In PLC/PRF/5 and Huh7 cells, we confirmed the inhibition of MEK by AZD6244 (0.1 µM for PLC/PRF/5 cells and 1 µM for Huh7 cells), AKT by MK2206 (0.1 µM for PLC/PRF/5 cells and 1 µM for Huh7 cells), and PI3K and mTOR by BEZ235 (0.01 µM for PLC/PRF/5 cells and 0.1 µM for Huh7 cells) based on significant decreases in the basal levels of phosphorylated extracellular-signal-regulated kinase (ERK), AKT, and p70S6kinase1, respectively (Fig. 4a). However, pretreatment with AZD6244, MK2206, and BEZ235 did not prevent TGF-β1-mediated xCT repression. The levels of phosphorylated ERK, AKT, or p70S6K1 were not significantly changed at 24 h after treatment with TGF-β1.

The basal and TGF-β1-inducible activities of p38 MAPK, JNK, and RhoA/ROCK as evidenced by the phosphorylation levels of p38 MAPK, c-Jun, and myosin phosphatase target subunit 1 (MYPT1), respectively, were not detected in these cells. TGF-β1 retained the ability to inhibit xCT expression following the inhibition of p38 MAPK by 5 µM of SB203580, JNK by 10 µM of SP600125, and ROCK1 by 10 µM of Y27632 (Fig. 4b). Independent of TGF-β1-mediated regulation, the basal levels of xCT expression were decreased by BEZ235 alone in Huh7 cells but increased by SP600125 alone in PLC/PRF/5 and Huh7 cells.

### Repression of xCT by TGF-β1 contributed to changes in ROS and GSH levels

**Fig. 5:**
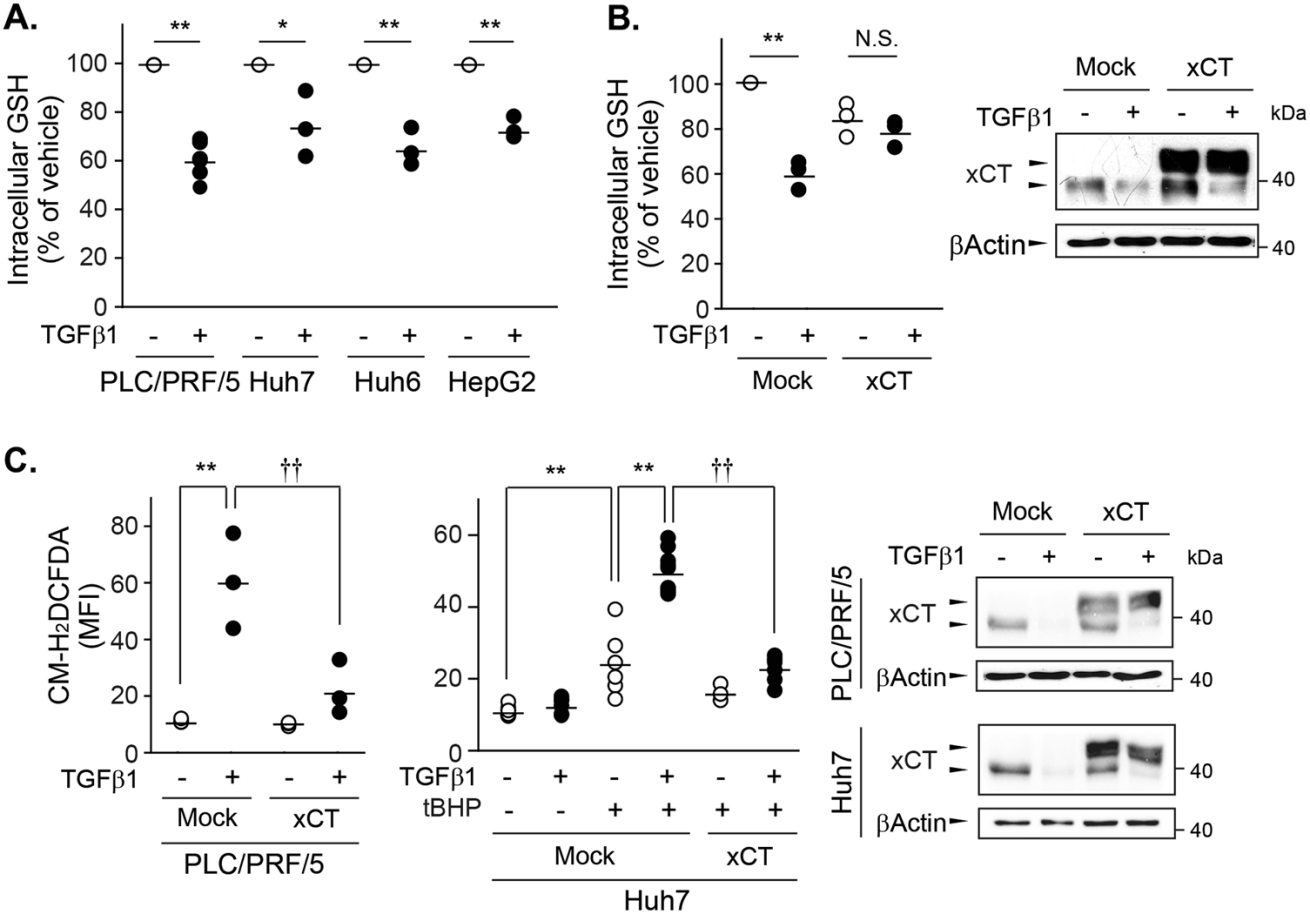
Changes in ROS and GSH levels due to TGF-β1-mediated xCT repression. **a** PLC/PRF/5 (*n* = 6), Huh7 (*n* = 3), Huh6 (*n* = 3), and HepG2 (*n* = 3) cells were treated with either vehicle or 5 ng/mL of TGF-β1 for 24 h and then collected to measure GSH levels. **b** PLC/PRF/5 cells (*n* = 3) transiently transfected with a mock- or Myc-DDK-tagged xCT-expressing vector were treated with vehicle or 5 ng/mL of TGF-β1 for 24 h and then sampled to measure GSH and protein expression levels. **c** PLC/PRF/5 (*n* = 3) and Huh7 (*n* = 3 or 6) cells transfected with a mock- or xCT-expressing vector were treated with 5 ng/mL of TGF-β1 for 24 h and then collected for ROS measurement using CM-H_2_DCFDA and western blot analysis. At 1 h before ROS measurement, 30 µM of tBHP was added to stimulate ROS generation in Huh7 cells. Overexpressed xCT was detected as having a higher molecular weight than endogenous xCT in the western blot analyses; multiple analyses are shown as the mean ± SD. *P < 0.05, **P < 0.01, TGF-β1 versus vehicle control; ††*P* < 0.01, TGF-β1 in mock-transfected cells versus TGF-β1 in xCT-transfected cells.

Decreases in xCT expression lead to a limited supply of l-cystine, which is required for GSH synthesis. The intracellular levels of GSH in PLC/PRF/5, Huh7, Huh6, and HepG2 cells were significantly decreased by treatment with 5 ng/mL of TGF-β1 for 24 h (Fig. 5a). The reductions in GSH levels were the largest in PLC/PRF/5 cells (41.2%) but more modest in Huh7 cells (26.4%). PLC/PRF/5 cells overexpressing xCT tagged with DDK did not exhibit TGF-β1-mediated reductions in GSH levels (Fig. 5b).

Next, we investigated whether the TGF-β1-induced decreases in GSH levels contributed to TGF-β1-induced ROS generation using chloromethyl-2′,7′-dichlorodihydrofluorescein diacetate (CM-H_2_DCFDA) dye uptake. Treatment with 5 ng/mL of TGF-β1 for 24 h increased ROS levels by 5.7-fold in mock-transfected PLC/PRF/5 cells but this increase was significantly blunted in xCT-overexpressed PLC/PRF/5 cells (Fig. 5c). In Huh7 cells, TGF-β1 alone did not increase ROS levels despite the occurrence of xCT repression. Given the limited changes in GSH contents and ROS generation by TGF-β1 in Huh7 cells, TGF-β1 might have induced mild oxidative stress that could be compensated by intracellular amount of GSH in this cell line. Because cancer cells may be exposed to high concentrations of ROS upon accelerated metabolism^27^, conditions of oxidative stress were mimicked in the Huh7 cells in the present study by exposure to 30 µM of tert-butyl hydroperoxide (tBHP) for 1 h. The Huh7 cells generated significant amounts of ROS following stimulation by tBHP, and the tBHP-initiated increase in ROS levels was enhanced by pretreatment of TGF-β1 for 24 h by 2.1-fold. However, the TGF-β1-mediated potentiation of ROS generation was not observed in xCT-overexpressing Huh7 cells consistently with the results in PLC/PRF5 cells.

### TGF-β1 potentiated lipid peroxidation

**Fig. 6:**
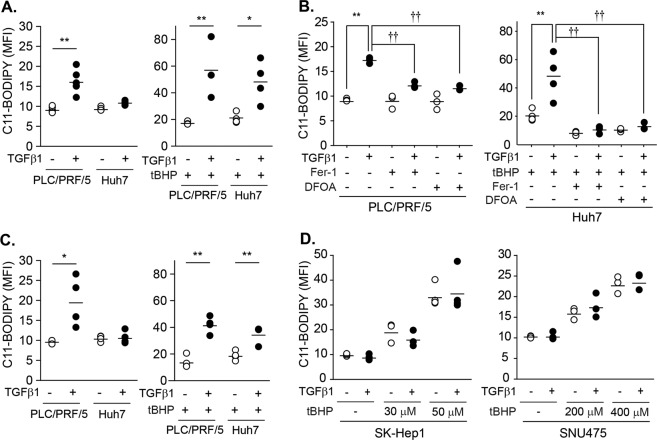
Potentiation of the tBHP-triggered lipid peroxidation by TGF-β1 in PLC/PRF/5 and Huh7 cells. **a** PLC/PRF/5 (*n* = 6) and Huh7 cells (*n* = 4) were treated with either vehicle or 5 ng/mL of TGF-β1 for 2 days. At 1 h before the measurement, tBHP was added at doses of 100 µM to PLC/PRF/5 cells (*n* = 3) and 50 µM to Huh7 cells (*n* = 4)(right). **b** Cells (*n* = 3) were pretreated with 20 µM Fer-1 1 h before TGF-β1 treatment. DFOA was added at doses of 100 µM 1 h before the measurement in PLC/PRF/5 cells or before tBHP treatment in Huh7 cells. TGF-β1 and tBHP were treated as described in **a**. The first two groups in Huh7 cells (tBHP, TGF-β1 with tBHP) are identical to **a** as these experiments were performed at once, but displayed again to enhance understanding. **c** PLC/PRF/5 (*n* = 4) and Huh7 cells (*n* = 4) were treated with either vehicle or 5 ng/mL TGF-β1 for 8 days. tBHP was added 1 h before measurement at doses of 100 µM in PLC/PRF/5 cells (*n* = 4) and 50 µM in Huh7 cells (*n* = 4). **d** SK-Hep1 (*n* = 3) and SNU475 (*n* = 3) cells were treated with 5 ng/mL of TGF-β1 for 2 days and then further incubated with tBHP at the indicated concentrations for 1 h. Lipid peroxidation was measured with the BODIPY® C11 probe and expressed as the mean fluorescence intensity (MFI); multiple analyses are shown as the mean ± SD. Representative histograms were shown in supplemental Fig. 1. *P < 0.05, **P < 0.01, TGF-β1 versus vehicle; †*P* < 0.05, ††*P* < 0.01, TGF-β1 in vehicle-treated cells versus TGF-Β1 in Fer-1- or DFOA-treated cells.

The inhibition of system x_c_^−^ promotes the iron-mediated lipid peroxidation and ultimately induces ferroptotic cell death^4^. Therefore, we assessed the effects of TGF-β1 on lipid peroxidation with the C11-BODIPY probe. In PLC/PRF/5 cells, lipid peroxidation levels significantly increased by 1.75-fold at 48 h after treatment with 5 ng/mL of TGF-β1 (Fig. 6a left). As the increase in lipid peroxidation in Huh7 cells was marginal after treatment with TGF-β1 for 48 h, we examined whether TGF-β1 enhanced tBHP-triggered lipid peroxidation. Based on preliminary analyses, 1 h of treatment with tBHP at doses of 100 µM in PLC/PRF/5 cells and 50 µM in Huh7 cells induced mild increases in lipid peroxidation levels (Fig. 6a right). The degree of lipid peroxidation triggered by tBHP was higher in the TGF-β1-pretreated group than the vehicle-pretreated group by 3.31-fold in PLC/PRF/5 cells and 2.34-fold in Huh7 cells. However, the increases in lipid peroxidation by TGF-β1 in PLC/PRF/5 cells and by TGF-β1 with tBHP in Huh7 cells were prevented by pretreatment with ferrostatin-1 (Fer-1), which is a lipophilic antioxidant, and deferoxamine (DFOA), which is an iron chelator (Fig. 6b). Cell viability following TGF-β1 treatment was not changed by the presence of these inhibitors under this condition (38 ± 8% in the control group; 33 ± 10% in the Fer-1-pretreated group; and 31 ± 3% in the DFOA-pretreated group). PLC/PRF/5 and Huh7 cells treated with TGF-β1 for 8 days still showed higher degrees of lipid peroxidation following the tBHP challenge (Fig. 6c). In contrast, TGF-β1 did not induce changes in basal or tBHP-triggered lipid peroxidation levels in SK-Hep1 or SNU475 cells, which have a late TGF-β1 gene signature (Fig. 6d). Taken together, these results demonstrate that the TGF-β1-provoked redox imbalance directly induced and enhanced lipid peroxidation in PLC/PRF/5 and Huh7 cells, respectively.

### GPX4 is a druggable vulnerability in TGF-β1-treated PLC/PRF/5 and Huh7 cells

**Fig. 7:**
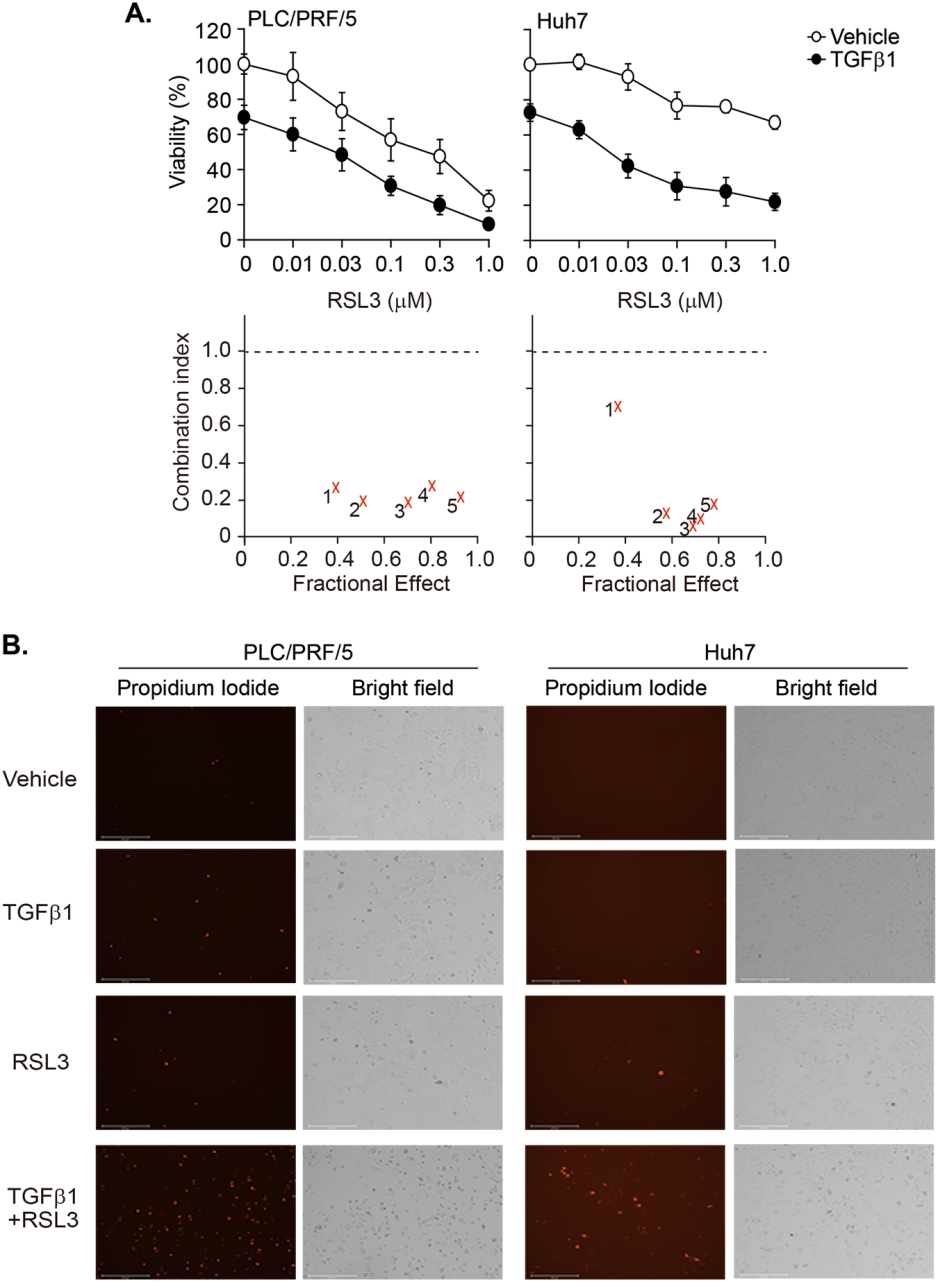
TGF-β1-mediated vulnerability to GPX4 inhibition in PLC/PRF/5 and Huh7 cells. Cells were incubated with vehicle or TGF-β1 (0.03 ng/mL for PLC/PRF/5 cells and 5 ng/mL for Huh7 cells) for 2 days. Then, the cells were exposed to RSL3 in a dose-dependent manner in the presence or absence of TGF-β1 for another 2 days. **a** Cell viability was measured with the CellTiter Glo® assay, and the CI was calculated following the Chou–Talalay method. 1~5 corresponds to the data for RSL3 dose at 0.01 (1), 0.03 (2), 0.1 (3), 0.3 (4), and 1.0 (5) µM. **b** Cell images were captured 1 day after treatment with 0.03 µM of RSL3. The cells were stained with 1 µg/mL PI for 20 min and then monitored with the EVOS® cell imaging system.

Lipid peroxidation end products are reduced into corresponding alcohols by GPX4 with the use of GSH^5^. Therefore, we hypothesized that the TGF-β1-induced sensitization to lipid peroxidation could be exploited to discover vulnerabilities to a GPX4 inhibitor in HCC cells. To accomplish this, TGF-β1 was applied to PLC/PRF/5 cells and Huh7 cells for 2 days, RSL3 was added in a dose-dependent manner, and further incubated for another 2 days. Because TGF-β1 alone decreased viability more than 50% in PLC/PRF/5 cells from the doses of 0.1 ng/ml, TGF-β1 was applied at the concentration of 0.03 ng/mL in PLC/PRF/5 cells and at 5 ng/ml in Huh7 cells to determine synergy. RSL3-mediated decreases in cell viability were higher in TGF-β1-pretreated cells than vehicle-pretreated cells (Fig. 7a). Combination index (CI) analyses revealed strong synergy across most ranges of the fractional effects in both cell lines. Permeabilization of plasma membrane and shrinkage of cells were observed 24 h after RLS3 treatment (Fig. 7b). Therefore, PLC/PRF/5 cells and Huh7 cells were vulnerable to RSL3 in the presence of TGF-β1.

## Discussion

Although it is commonly accepted that TGF-β1 increases oxidative stress^19^, the role of TGF-β1 in the regulation of system x_c_^−^ has yet to be fully elucidated. We identified xCT as a target gene that was regulated by TGF-β1 in well-differentiated HCC cells that exhibited cytostasis in response to TGF-β1 treatment. This decreased xCT expression contributed to TGF-β1-induced depletions of GSH, increases in ROS production, and ultimately sensitize cells toward lipid peroxidation.

The present study demonstrated that TGF-β1 regulated xCT expression in PLC/PRF/5, Huh7, Huh6, and HepG2 cells, which have an early TGF-β1 gene signature. This type of cell line exhibits cytostasis and apoptosis in response to TGF-β1 with strong transcriptional Smad3 activity^24,25^. Therefore, the mechanisms underlying the repression of xCT by TGF-β1 were likely associated with a Smad3-dependent gene signature. The accumulation of genetic alterations in tumors drives TGF-β1 to exert tumor-promoting activities^26^. The present results showed that TGF-β1 had little effect on Smad3 and xCT expressions in SNU387, SNU449, SNU475, and SK-Hep1 cells even though TGF-β1 increased Smad2/3 phosphorylation levels. These cells are classified as having a late TGF-β1 gene signature that is associated with high levels of TGF-β1, a lack of TGF-β receptors, reduced Smad3 signaling, and high levels of EMT-associated proteins^24,25^. Thus, the selective decapitation of core TGF-β1 components for the purposes of tumor suppression could be associated with the loss of control for xCT expression by TGF-β1 in these cell types.

The context-dependent regulation of gene expression by Smads in TGF-β1 signaling has been well characterized^18,26^. Some genes are redundantly regulated by Smad2 and Smad3, whereas other genes are exclusively regulated by Smad3, which occurs with c-myc and programmed death-1 (PD-1)^28–30^. Smad3-mediated tissue-specific gene expression is regulated by interactions with other transcription factors, such as thyroid transcription factor-1 (TTF-1) in lung epithelial cells^31^. The present findings following chemical inhibition, gene knockdown, or gene overexpression indicated that Smad3 predominantly regulated xCT expression in the absence of cooperation with Smad2, Smad4, and non-Smad signaling. Smad3 might interact with other proteins that are specific to these type of cells to control xCT expression. In the present study, TGF-β1 greatly reduced xCT protein levels and partially suppressed xCT mRNA levels, which indicated that the mechanisms underlying this process involved transcription-dependent and -independent regulation systems. Although results from the Pscan software tool (OmicX) suggested that there were two putative Smad3 binding sites in the xCT promoter region, TGF-β1-inducible Smad3 binding was not readily observed using chromatin immunoprecipitation assays in our preliminary study. Smad3 might also decrease xCT protein stability via ubiquitination and degradation, as with the control of the vascular endothelial growth factor A (VEGFA) protein^32^. The present study also tried to investigate the effects of TGF-β1 on xCT protein stability, but TGF-β1 did not decrease xCT expression following treatment with cycloheximide (data not shown), which suggests that novel protein synthesis, such as Smad3, would be required for this type of regulation.

The present study demonstrated that xCT repression was one reason underlying the TGF-β1-mediated redox imbalance by the fact that the overexpression of xCT prevented ROS generation induced by TGF-β1 in PLC/PRF/5 cells or induced by TGF-β1 and tBHP in Huh7 cells. Considering that basal levels of oxidative stress may vary across different cancer cells and higher metabolic need in mitochondria could promote oxidative stress, a tBHP challenge could mimic this pathophysiological situation. The molecular events underlying the propagation of cytosolic ROS to induce membrane peroxidation and the execution of ferroptosis by lipid peroxidation have yet to be fully elucidated^2^. In the present study, prolonged exposure to TGF-β1 for 48 h directly induced lipid peroxidation or potentiated the tBHP-initiated lipid peroxidation. Accumulation of lipid peroxidation by TGF-β1 was a late cellular event and likely to be non-lethal, because the decreases in cell viability by TGF-β1 was not influenced by Fer-1 (Fig. 6b, results section). As TGF-β1 activates multiple pathways involved in growth arrest and apoptosis, including p21, Bcl-xL, and Bim^33,34^, the rescue from ferroptotic stress by Fer-1 could not revert the initial pleotropic actions of TGF-β1.

The present findings revealed a novel role of TGF-β1 in terms of rendering well-differentiated HCC cells vulnerable to a GPX4 inhibitor. Although GPX4 is a promising pharmacological target for the induction of ferroptosis in cancer cells, there is a great need in terms of identifying tumors that might benefit from treatment with a GPX4 inhibitor^2,5^. This study found that RSL3-induced cell death was higher in cells pretreated with TGF-β1 than in control cells. Because median serum TGF-β1 levels in HCC patients are approximately 13.7 ng/mL^35^, it is relevant that TGF-β1 secreted from tumor microenvironment can induce xCT repression and enhance GPX4 inhibitor-mediated cell death in these HCC cells. As GPX4 inhibitor having appropriate pharmacokinetic properties for in vivo study are currently in development^2^, further studies employing GPX4 inhibitor in orthotopic tumor models with TGF-β1-rich microenvironments may support our hypothesis. Recent transcriptome analyses using open access data and HCC cases have identified a cluster of HCC cases that exhibit activation of the TGF-β1 signaling pathway, which is positively associated with a favorable prognosis^36^. The active TGF-β1 signaling in this patient population may correspond to that of HCC cells with an early TGF-β1 gene signature. The present findings suggest that TGF-β1 may selectively regulate xCT expression in these types of tumors, which may benefit from treatment with a GPX4 inhibitor.

In conclusion, the present findings provide the first evidence that TGF-β1/Smad3 play a role in the repression of system x_c_^−^ activity and promotes lipid peroxidation to a sublethal or mild degree, rendering sub-populations of HCC cells to vulnerable to GPX4 inhibition.

## Materials and methods

### Cell culture and treatment

The PLC/PRF/5 cell line was purchased from the American Type Culture Collection (Manassas, VA, USA), the Huh6 and HepG2 cell lines were supplied by the RIKEN Bioresource Research Center (Tsukubu, Japan), and the Huh7, SNU387, SNU449, SNU475, and SK-Hep1 cell lines were obtained from the Korean Cell Line Bank (Seoul, Korea). Huh7, Huh6, and HepG2 cells were cultured in Dulbecco’s modified Eagle medium (Thermo Fisher Scientific, Waltham, MA, USA) and the other cell lines were grown in RPMI-1640 medium (Thermo Fisher Scientific). The cells were maintained in medium supplemented with 10% fetal bovine serum (FBS), 2 mM l-glutamine, 100 U/mL penicillin, and 100 µg/mL streptomycin at 37 °C in a humidified 5% CO_2_ incubator, and were regularly assessed to confirm the absence of mycoplasma contamination, as previously described^37^. Recombinant human TGF-β1 (R&D Systems, Minneapolis, MN, USA) was added to the cultures 18 h after pre-incubation in medium containing 2% FBS; the TGF-β1 and media were replaced every 3 days to prevent depletion if needed.

AZD6244, MK2206, and BEZ235 were obtained from Selleckchem (Houston, TX, USA), SB203580 and SP600125 were purchased from Enzo Life Sciences (Farmingdale, NY, USA), and SB431542 and Y27632 were supplied by MilliporeSigma (Burlington, MA, USA); these inhibitors were administered 1 h before TGF-β1 treatment. RSL3 was supplied by Selleckchem, and PI was obtained from MilliporeSigma. In the control group, phosphate-buffered saline (PBS) and dimethyl sulfoxide were used as vehicle treatments for TGF-β1 and the other inhibitors, respectively.

### Cell viability

Cell viability was measured using the CellTiter Glo® assay (Promega, Madison, WI, USA) according to the manufacturer’s instructions. Cells were seeded in fresh medium in a 96-well plate at a density of 2000 cells per well and then, at 24 h after seeding, were exposed to vehicle or TGF-β1 for 6 days. Luminescence was measured with an EnVision® multimode plate reader (PerkinElmer, Waltham, MA, USA) and synergistic effects were measured with CalcuSyn software (Biosoft, Palo Alto, CA, USA) using the Chou–Talalay method^38^. CI values of 0.1–0.3, 0.3–0.7, and 0.7–0.85 indicated strong synergism, synergism, and moderate synergism, respectively. All cell images were captured with the EVOS® cell imaging system (Thermo Fisher Scientific).

### Protein sampling and western blot analysis

Whole-cell lysates were prepared with lysis buffer, as previously described^37^. Cell fractions of the plasma membrane and cytosol were isolated with the Mem-PER™ Plus Membrane Protein Extraction Kit (Thermo Fisher Scientific) according to the manufacturer’s instructions and protein concentrations were determined using the Bradford protein assay (Bio-Rad, Hercules, CA, USA). Then, 15-µg protein samples were resolved using sodium dodecyl sulfate polyacrylamide gel electrophoresis on either 8% or 12% gels, transferred onto nitrocellulose membranes, probed with primary and secondary antibodies, and detected with a horseradish peroxidase substrate (Thermo Fisher Scientific) using the iBright CL1000 Imaging System (Thermo Fisher Scientific). The following primary antibodies were used: xCT (#1269), phospho-Smad2 (Ser 465/467; #8828), phospho-Smad3 (Ser 423/425; #9520), Smad2/Smad3 (#5678), Smad4 (#3845), E-cadherin (#3195), phospho-ERK (#4370), ERK (#4696), phospho-AKT (#4060), AKT (#2920), and phospho-p70S6-kinase1 (#9205) were purchased from Cell Signaling Technology (Danvers, MA, USA); p70S6-kinase1 was supplied by Epitomics (Burlingame, CA, USA); and anti-4F2hc (sc-390154), anti-HSP70 (sc-66048), and anti-β-actin (sc47778) were obtained from Santa Cruz Biotechnology (Santa Cruz, CA, USA). Horseradish peroxidase-linked secondary antibodies were obtained from Jackson ImmunoResearch (West Grove, PA, USA). All antibodies were diluted to between 1:2000 and 1:10,000.

### Gene expression analysis

Total RNA was extracted using TRIzol® reagent (Invitrogen, Carlsbad, CA, USA) according to the manufacturer’s instructions and then 2-µg aliquots were reverse-transcribed in a mixture containing AMV Reverse Transcriptase (Promega, Madison, WI, USA), deoxynucleoside triphosphates, and oligo(dT)_16_. The resulting cDNA was amplified with a real-time polymerase chain reaction (RT-PCR) reaction with QuantStudio3 using *Power* SYBR™ Green PCR Master Mix (Thermo Fisher Scientific) according to the manufacturer’s instructions. The following primers were supplied from Bioneer (Daejeon, Korea): human xCT, 5′-ATGGTCAGAAAGCCTGTTGT-3′ (sense); 5′-TAGTGACAGGACCCCACACA-3′ (antisense); human vimentin, 5′-CAGGCAGAGAATGCTGAGTTC-3′ (sense); 5′-CATCACCAGCTTAAAGCCTT-3′ (antisense); human β-actin, 5′-AGCGGGAAATCGTGCGTG-3′ (sense); and 5′-CAGGGTACATGGTGGTGCC-3′ (antisense). After amplification, a melting curve analysis was performed to verify the specificity of the amplicon and the relative quantification was analyzed using the ΔΔCT method.

### Transfection

For the transient knockdowns, cells at 50–60% confluence in opti-MEM medium (Thermo Fisher Scientific) were transfected with DharmaFECT reagent (Dharmacon, Lafayette, CO, USA) using 100 ng of small interfering RNA (siRNA) that targeted Smad2, Smad3, Smad4, or a scrambled control siRNA (Genolution, Seoul, Korea). For transient transfection, cells were transfected with pCMV5B-Flag-Smad3 (Addgene, Watertown, MA, USA), pCMV6-Myc-DDK-tagged SLC7A11 (OriGene, Rockville, MD, USA), or a corresponding control plasmid using lipofectamine 3000 (Thermo Fisher Scientific). After 3 h of transfection, the cells were recovered in medium containing 2% FBS for 24 h before TGF-β1 treatment.

### Measurement of redox status

Intracellular ROS and lipid peroxidation levels were assessed after treatment with TGF-β1 in the presence or absence of tBHP; tBHP (MilliporeSigma) concentrations in each cell line were preliminary evaluated to ensure that oxidative stress was appropriately triggered. Intracellular ROS levels were detected with cell-permeant CM-H_2_DCFDA (Thermo Fisher Scientific). After treatment with TGF-β1 and/or tBHP, the cells were washed and exposed to pre-warmed PBS containing CM-H_2_DCFDA for 30 min. Lipid peroxidation was detected with the Image-iT™ Lipid Peroxidation Kit based on the lipophilic BODIPY® 581⁄591 C11 probe (Thermo Fisher Scientific). After treatment with TGF-β1 and/or tBHP, the BODIPY® probe was added and cells were incubated for 30 min at 37 °C. The cells were collected via trypsinization, washed with PBS, and then fluorescence was detected using a Guava® easyCyte flow cytometer (MilliporeSigma) with excitation/emission at 488/525 nm; the results were analyzed using InCyte2.6 software (MilliporeSigma).

Intracellular GSH levels were determined using the Glutathione Fluorometric Assay Kit (BioVision, Milpitas, CA, USA) according to the manufacturer’s instructions. Briefly, 1 × 10^6^ cells were collected and precipitated with 6N perchloric acid. Next, the supernatant was neutralized with 3 N KOH, diluted with an assay buffer, and incubated with an *o*-phthalaldehyde probe for 40 min. Fluorescence levels with excitation/emission at 340/420 nm were measured with an Infinite® 200 microplate reader (Tecan, Männedorf, Switzerland). The relative amount of GSH was calculated as the ratio of the TGF-β1-treated group to the vehicle-treated group.

### Statistical analysis

All data are included, if positive control worked or obvious mistake was not happened, and expressed as the mean ± standard deviation (SD); ‘*n*’ indicates the number of independent in vitro experiments for a particular set of experiments. One-way analysis of variance and Student’s *t*-test were used for data comparisons. All statistical analyses were conducted using SPSS for Windows software (ver. 22; IBM Corp., Armonk, NY, USA) and *P* values < 0.05 were considered to indicate statistical significance.

## Supplementary information

Supplementary Figure 1

Supplementary Figure Legends
