# Supplementary figures and images for: TGF-β1-mediated repression of SLC7A11 drives vulnerability to GPX4 inhibition in hepatocellular carcinoma cells
Source: Cell Death Dis. 2020 May 29;11(5):406. doi: 10.1038/s41419-020-2618-6 (PMC7260246; doi:10.1038/s41419-020-2618-6)

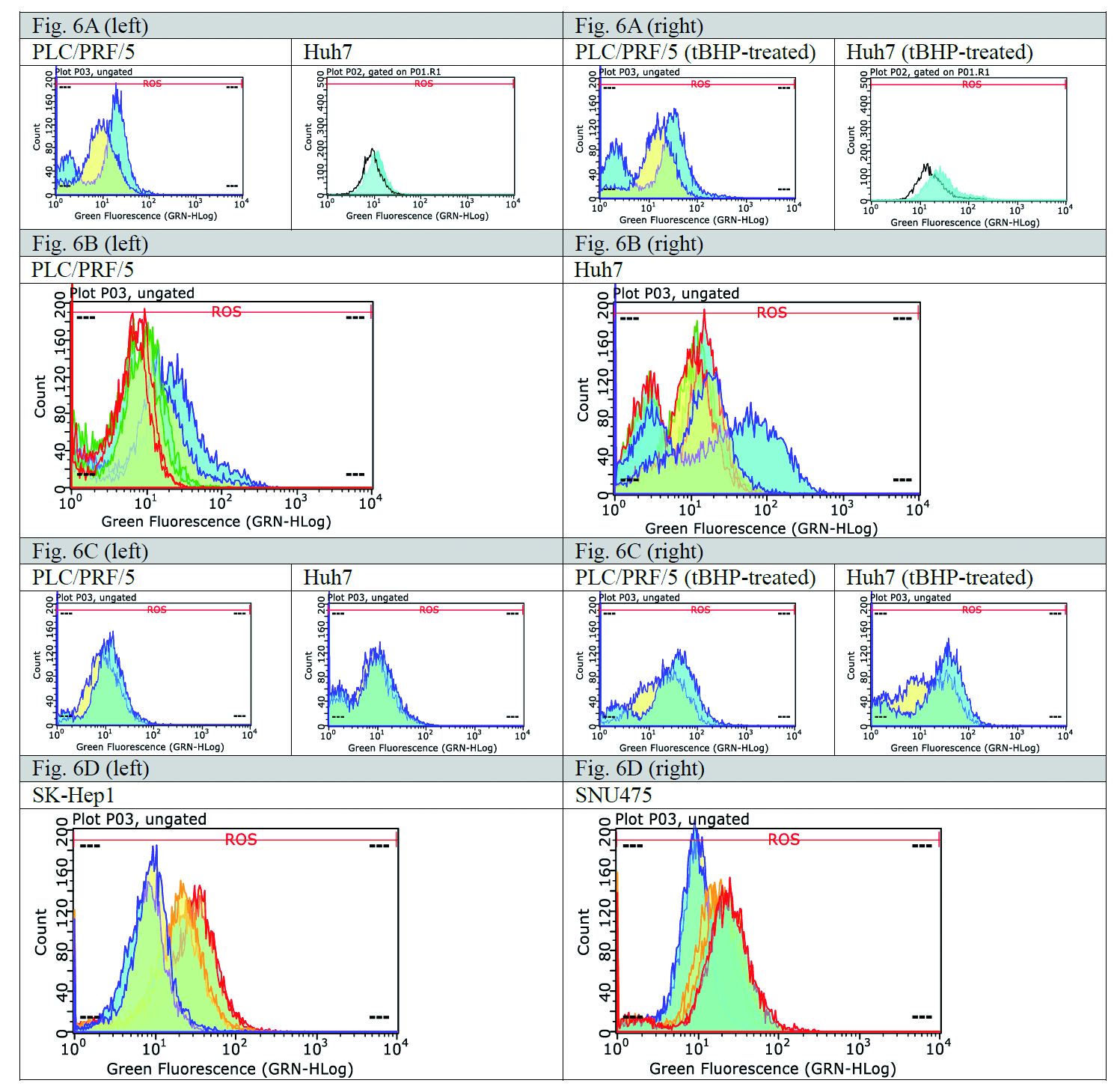

Supplement: Supplementary file 1 — Supplementary Figure 1 [file 41419_2020_2618_MOESM1_ESM.tif]
