# Supplementary material for: TGF-β1-mediated repression of SLC7A11 drives vulnerability to GPX4 inhibition in hepatocellular carcinoma cells
Source: Cell Death Dis. 2020 May 29;11(5):406. doi: 10.1038/s41419-020-2618-6 (PMC7260246; doi:10.1038/s41419-020-2618-6)
Supplement: Supplementary file 2 — Supplementary Figure Legends [file 41419_2020_2618_MOESM2_ESM.docx]

**Supplemental figure 1.** **Representative histograms of Figure 6.** Representative histograms for the detection of lipid peroxidation by BODIPY^®^ 581⁄591 C11 probe in Figure 6 were shown. Yellow or white field, vehicle-treated; Blue field, TGFβ1-treated: Fig. 6B, Blue line, vehicle-treated; Green line, deferoxamine-treated; Red line, Ferrostatin-1-treated: Fig. 6D, Blue line, vehicle-treated; Orange line, 30 μM (SK-HEP-1) or 200 μM (SNU475) tBHP-treated; Red line, 50 μM (SK-HEP-1) or 400 μM (SNU475) tBHP-treated.
